# SOX6 blocks the proliferation of BCR-ABL1^+^ and JAK2V617F^+^ leukemic cells

**DOI:** 10.1038/s41598-019-39926-4

**Published:** 2019-03-04

**Authors:** Gloria Barbarani, Cristina Fugazza, Silvia M. L. Barabino, Antonella E. Ronchi

**Affiliations:** Dipartimento di Biotecnologie e Bioscienze, Università degli Studi di Milano-Bicocca, Piazza della Scienza 2, 20126 Milano, Milano Italy

## Abstract

SOX6 is a HMG-box transcription factor expressed in a wide range of tissues. Recent data show that SOX6 expression is altered in different cancers, in the majority of cases being downregulated. To date, no data are available about SOX6 role in hematological malignancies. Here we demonstrate that SOX6 overexpressing BCR-ABL1^+^ B-ALL cells are unable to promote leukemia in a mouse model. Starting from this observation, we extended our study to a panel of human leukemic cells carrying genetic lesions distinctive of different types of leukemias and myeloproliferative disorders (the BCR-ABL1 translocation and the JAK2V617F amino acid substitution) to dissect the cellular events induced by SOX6. The inhibition of proliferation is the invariant outcome of SOX6 overexpression but it is achieved via two different cellular responses: terminal differentiation in erythroid-biased cells, irrespectively of their mutation, and apoptosis in megakaryocytic-primed and lymphoid cells. Within this context, cells carrying the highest copy number of the JAK2V617F allele better counteract the SOX6-imposed growth arrest. The interrogation of the GEPIA (Gene Expression Profiling Interactive Analysis) human dataset reveals that *SOX6* is downregulated in a cohort of AML patients, uncovering a wide anti-proliferative role of SOX6 in a variety of mutant backgrounds.

## Introduction

The SOX6 transcription factor, belonging to the Sry-related HMG-box family, is expressed in several tissues during development, where it plays a key role in the transition from proliferating progenitors to functionally mature cells^1^. In the adult, its role is more elusive.

Data collected in recent years suggest that *SOX6* expression is deregulated in different cancers. Often, SOX6 seems to act as tumor suppressor such as in Esophageal Squamous Cell Carcinoma -ESCC-^2^, Hepatocarcinoma -HCC-^3,4^, ovarian cancer^5^, pancreatic cancer^6^, colorectal cancer^7^ and its downregulation is frequently associated with a poor prognosis^2,4,5^. Rescue experiments performed by SOX6 overexpression in these types of cancers often result in a mitigated malignant phenotype^2,5,8,9^. In contrast, in other cancers, such as some brain tumors^10^, *SOX6* expression is higher compared to normal tissues. The cause of *SOX6* deregulation and, ultimately, the alteration of the cellular events downstream to it, remains largely unknown.

In hematopoiesis, SOX6 is required for proper erythroid terminal differentiation^11,12^. Its knock out in mouse results in compensated anemia^13^; its conditional ablation in erythroid cells impairs erythropoiesis in both homeostatic and stress condition^11^. Conversely, SOX6 overexpression induces a strong hemoglobinization, in both murine and human cellular model systems^12,13^. Interestingly, we noticed that SOX6 overexpression in cells carrying different genetic lesions, BCR-ABL1^+^ (e14a2 splicing variant p210^BCR-ABL1+^) (K562) and JAK2V617F^+^ (JAK2 c.1849G > T; p.Val617Phe) (HEL), leads to different proliferation kinetics. Indeed, HEL cells keep growing even when the K562 culture is already exhausted^12^.

The BCR-ABL1 fusion protein is the hallmark of Chronic Myeloid Leukemia (CML)^14,15^, but it is also a frequent cytogenetic abnormality in precursor B-lymphoblastic leukemia (B-Acute Lymphoblastic Leukemia, B-ALL) found in adults^16^ and, more rarely, in pediatric patients^17^. The JAK2V617F mutation is typical of myeloproliferative disorders (MPDs) and Acute Myeloid Leukemia (AML)^18^.

Here we show that SOX6 overexpression invariably blocks cell proliferation in both BCR-ABL1^+^ and JAK2V617F^+^ cells, although the *JAK2V617F* allele confers a graded resistance to SOX6-induced growth arrest depending on its copy number. The cellular mechanisms through which this is achieved is via terminal differentiation or apoptosis. Indeed, in both genetic backgrounds, SOX6 induces differentiation in erythroid-primed cells whereas it promotes apoptosis in erythro/megakaryocytic and B lymphoid cell types. We propose that the two aforementioned cellular pathways activated by SOX6 may account of its wide anti-proliferative role in different blood cell types. This is further supported by data recently made available^19^, showing that *SOX6* is downregulated in a cohort of AML patients.

## Results and Discussion

In recent years, several studies reported a reduced level of *SOX6* expression in different cancers. To explore its possible onco-suppressive role in hematological cancers, we first exploited the leukemia model of BCR-ABL1-induced B-ALL, generated in Prof. J. Ghysdael’s laboratory by transducing Bone Marrow cells from Cdkn2a-deficient C57BL/6J mice with a retroviral vector encoding for the e1a2 splicing variant of the p190^BCR-ABL1^ protein and GFP. Genomic deletions of the *Cdkn2a* locus are a frequent event in BCR-ABL1-positive ALL and are a prognostic marker for poor long-term outcome^20^. The presence of GFP allows the tracing of these cells (from here called B-ALL) in recipient mice.

**Figure 1:**
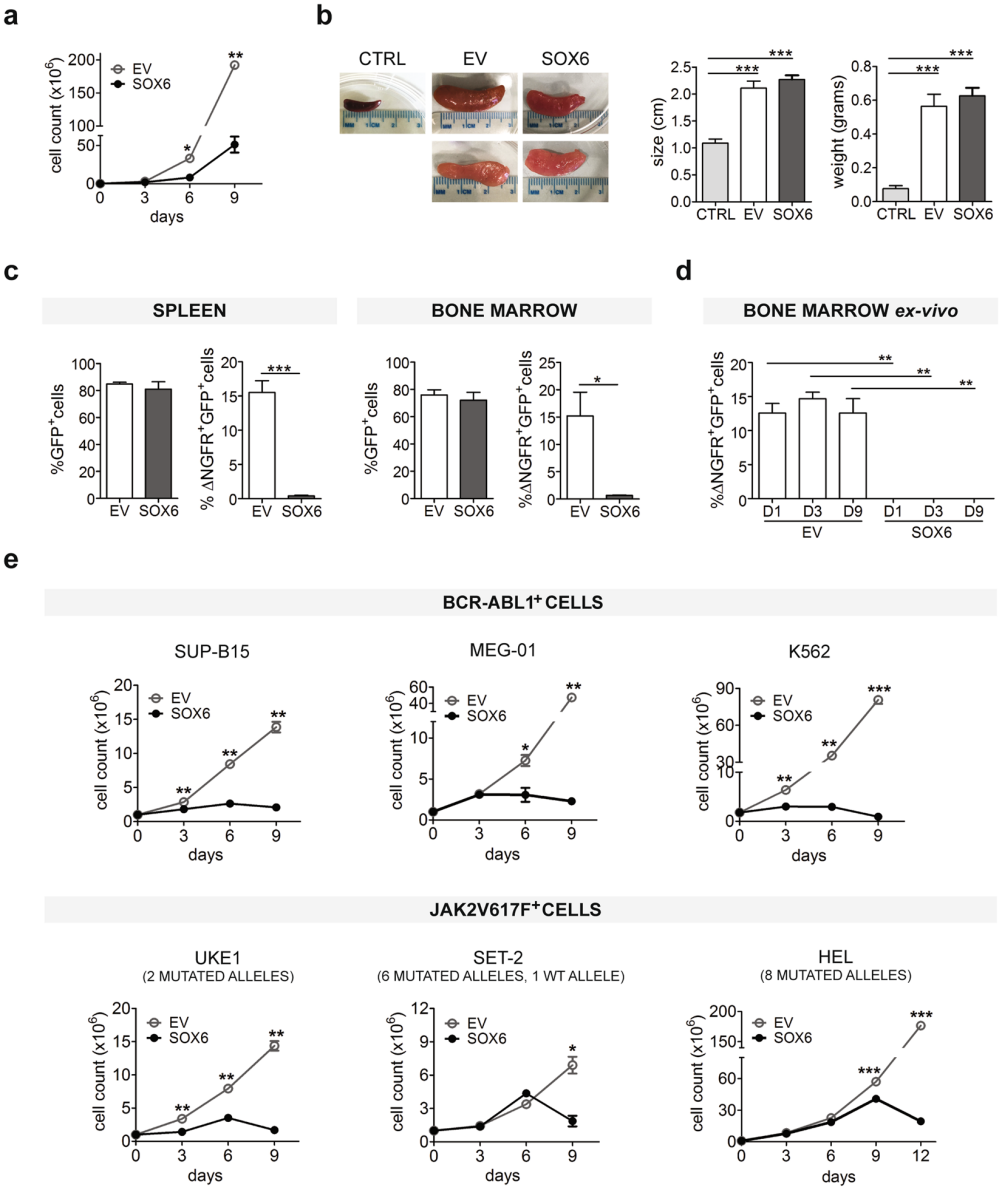
SOX6 blocks leukemia development. (**a**) Growth kinetics of B-ALL cells transduced either with viral particles carrying the SOX6-IRES-ΔNGFR (SOX6) or with EV-IRES-ΔNGFR (EV) vectors. (**b**) Analysis of spleens from mice injected with 10^6^ B-ALL transduced cells. Spleens were collected, measured, weighted (histogram bars: mean ± SEM n = 9; *p value < 0.05; **p value < 0.01; ***p value < 0.001) and compared to spleens from PBS-injected control animals (CTRL). (**c**) The transduced B-ALL engraftment contribution was assessed by tracking GFP^+^ cells (B-ALL cells) and GFP^+^ ΔNGFR^+^ double positive cells (B-ALL cells transduced with either SOX6 or EV) by flow cytometry (histogram bars: mean ± SEM; n = 9; *p value < 0.05; **p value < 0.01; ***p value < 0.001 P). (**d**) The same analysis was performed on explanted bone marrow cells grown *ex vivo* for 9 days (D1–D9) (histogram bars: mean ± SEM; n = 3). (**e**) Transduced cell lines growth curves. Transduced cells were ≥95% in each experiment, Fig. S1 (Graph points: mean ± SEM; n ≥ 3 *p value < 0.05; **p value < 0.01; ***p value < 0.001).

B-ALL were firstly transduced either with viral particles carrying the empty vector (EV) or the SOX6-ΔNGFR (SOX6) cassette. In each experiment, the efficiency of transduction was tested by western blot and flow cytometry (FC) (Fig. S1). In the infected cells, expanded in an *in vitro* culture, SOX6 overexpression induced a significant block in cell proliferation (Fig. 1a). We then injected 10^6^ cells, infected with a comparable efficiency for both EV and SOX6-ΔNGFR (not shown), into recipient age-matched C57BL/6J mice and monitored the development of leukemia (Fig. 1b,c). As expected, all mice developed leukemia within 9 days from transplantation and were sacrificed at the onset of first symptoms. Injected cells infiltrated in both spleen and bone marrow and represented about 80% of the total cells extracted from these organs, confirming that leukemia was indeed due to B-ALL-GFP^+^ cells (Fig. 1b,c). Strikingly, ΔNGFR-GFP double positive cells were about 15% in control mice injected with EV-transduced cells but less than 0.5% in mice injected with SOX6-tranduced cells (Fig. 1c). Furthermore, GFP^+^ ΔNGFR^+^ cells were absent in cultures expanded *ex vivo* from the bone marrows of mice injected with SOX6-transduced cells (Fig. 1d). Overall, these data provide a clear evidence that the anti-proliferative pathways activated by SOX6 counteract the leukemic potential of B-ALL cells, even in the presence of the double BCR-ABL1^+^ and *Cdkn2a*^−/−^ mutation, a combination that frequently is prognostic of a more aggressive lymphoid leukemia^21^. Moreover, these results suggest that SOX6 may act as an anti-leukemic factor in a *Cdkn2a*^−/−^ background. This evidence is particularly interesting since the p14ARF-HDM2-p53 axis is a known mediator of the SOX6 anti-proliferative effect in several tumors^8^. In order to extend this observation, we selected three human leukemic cell lines carrying the BCR-ABL1 translocation in different lineage background: SUP-B15, a B-cell precursor (e1a2 splicing variant p190^BCR-ABL1+^ CDKN2A^−/−^ ^22^); K562 and MEG-01, two erythro/megakaryocytic cell types (e14a2 splicing variant p210^BCR-ABL1+^ p53^−/−^ ^23^ and e13a2 splicing variant p210^BCR-ABL1+^ p53^−/−^ ^24^, respectively). As shown in Fig. 1e, upper panel, SOX6 overexpression invariably blocks cell proliferation in all the three cell types.

### The JAK2V617F mutation confers a graded resistance to the SOX6 anti-proliferative effect

In a previous work, we demonstrated that the erythroleukemic HEL cells behave differently compared to K562 upon SOX6 overexpression. Since HEL cells carry the JAK2V617F amino acid substitution, we hypothesized that the presence of this allele could counteract SOX6-induced growth arrest^12^.

To reinforce this hypothesis, we took advantage of human erythro/megakaryocytic leukemic cell lines carrying a different copy number of the *JAK2V617F* allele: HEL (8 copies)^25^, SET-2 (6 copies and one wt allele)^26^ and UKE1(2 copies)^27^.

As shown in Fig. 1e, lower panel, JAK2V617F^+^ cells indeed exhibited a graded resistance to the anti-proliferative SOX6 action depending on the mutant allele copy number. In fact, HEL cells overexpressing SOX6 grew at the same rate of control cells until day 6, started to decline at day 9, and the culture finally exhausted by day 12. SET-2 cells declined after day 6. Finally, in UKE1 cells, the SOX6 induced arrest in cell proliferation was already detectable at day 3.

### SOX6 induces apoptosis in non-erythroid cells

Since different cell types carrying contradistinctive genetic lesions stop growing upon SOX6 overexpression, we further investigated the cause of the proliferation block. We therefore analyzed -in both BCR-ABL1^+^ and JAK2V617F^+^ cells- three different parameters: i) the proportion of apoptotic cells (by AnnexinV and 7-AAD staining and subsequent FC analysis); ii) the induced differentiation (by testing the expression of prototypical lineage-specific genes); iii) the cells distribution across the different cell cycle phases (by PI staining and subsequent FC analysis).

**Figure 2:**
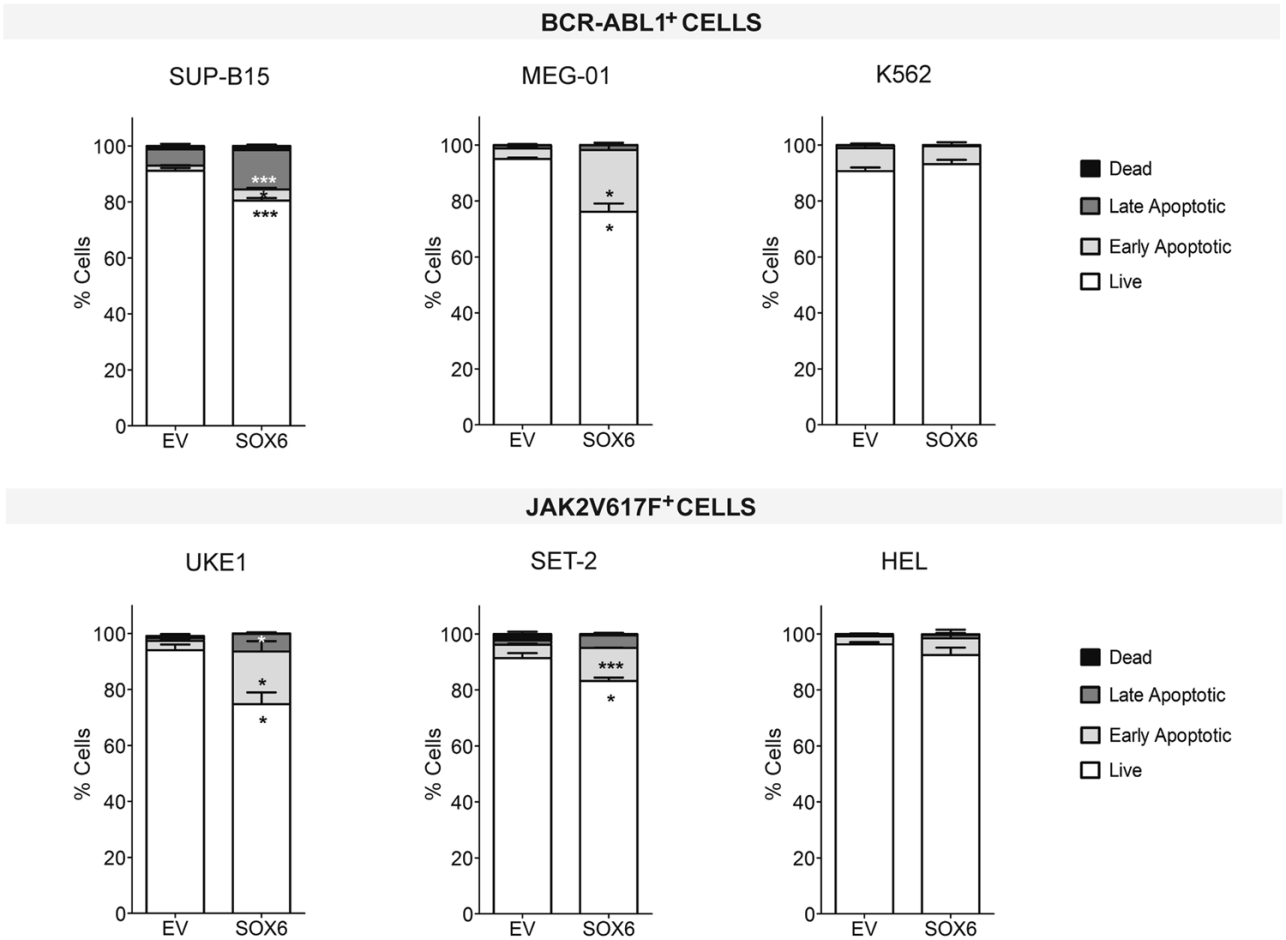
SOX6 induces apoptosis in non-erythroid biased cell lines. Apoptosis was assessed by staining transduced cells with AnnexinV and 7-AAD. Black: dead cells (AnnV^−^ 7-AAD^+^); dark grey: late apoptotic (AnnV^+^ 7-AAD^+^); light gray: early apoptotic (AnnV^+^ 7-AAD^−^); white: live cells (AnnV^−^ 7-AAD^−^) (histogram bars: mean ± SEM; n ≥ 3). Representative plots for each cell type are shown in Fig. S2.

As shown in Fig. 2 (upper panel), within the BCR-ABL1^+^ background, the percentage of SOX6-transduced cells undergoing apoptosis was significantly increased in MEG-01 and -more markedly- in SUP-B15 cells, compared to the EV-transduced control cells. In contrast, SOX6 overexpression did not promote apoptosis in K562 cells.

Instead, the analysis of JAK2V617F^+^ cells pointed to a graded resistance to apoptosis based on the different copy number of the mutant allele, similarly to what we observed for growth kinetics (Fig. 2, lower panel). In fact, whereas HEL cells, which carry 8 copies, did not show a significant increase of apoptotic cells in response to SOX6 overexpression, SET-2 (6 copies) and UKE1 (2 copies) proportionally increased the level of apoptotic cells.

### SOX6 induces differentiation in erythroid cells

SOX6 is involved in the production of mature red blood cells, being a key transcription factor required for the balance between cell cycle withdrawal and erythroid terminal differentiation. Since both K562 and HEL cells are commonly accepted as erythroid cellular model systems, we investigated to what extent the observed arrest in cell proliferation and the absence of apoptosis are linked to the erythroid differentiation program.

**Figure 3:**
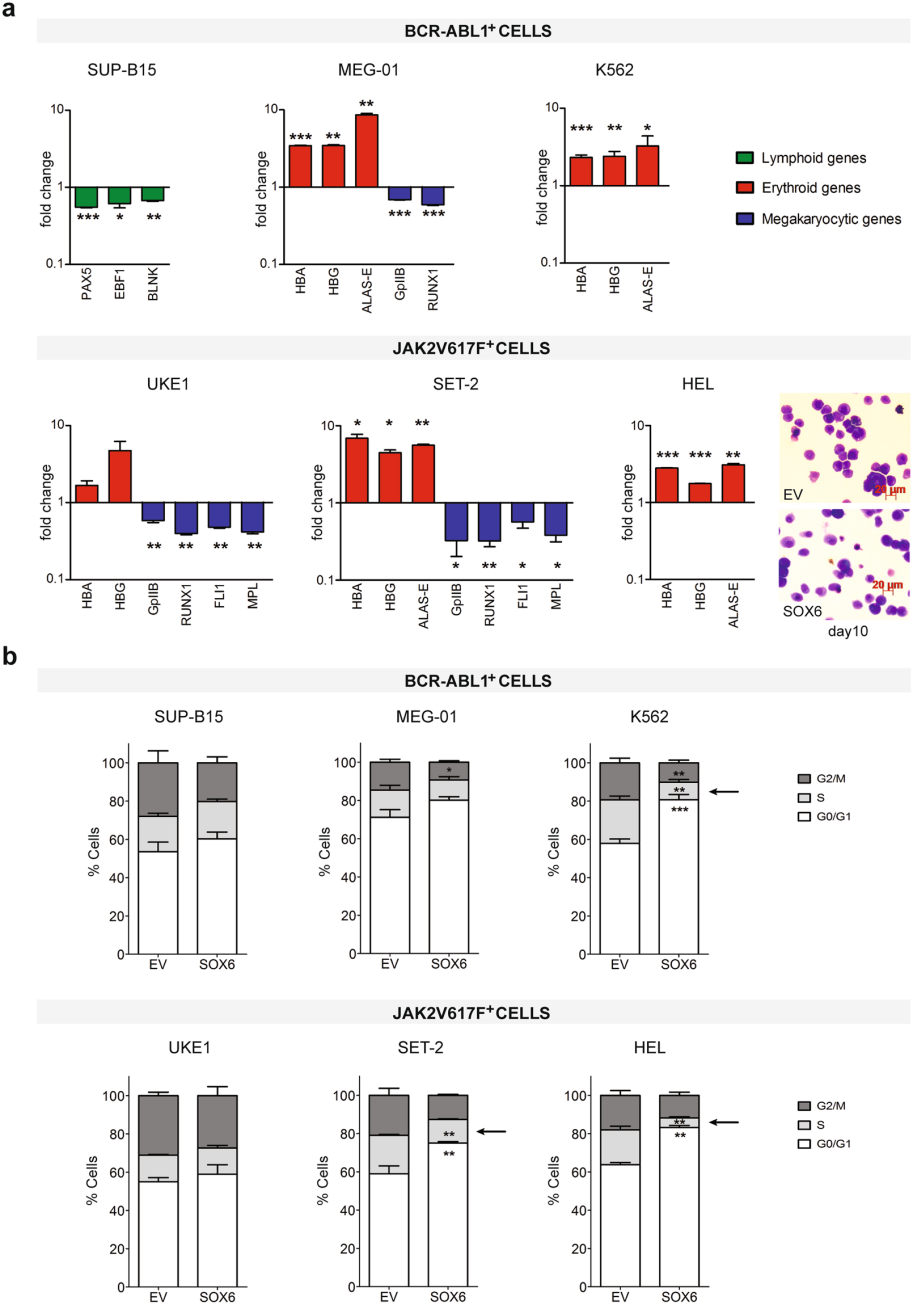
SOX6 induces erythroid genes at the expense of genes affiliated with other lineage programs. (**a**) Expression level of marker genes upon SOX6 transduction. Fold change variation in different lineage-specific genes (erythroid, megakaryocytic, lymphoid) was assessed by RT-qPCR (n ≥ 3). In the erythroleukemic HEL cells overexpressing SOX6 the enhanced erythroid differentiation was confirmed by cells staining. *HBA*: Hemoglobin α chain; *HBG*: Hemoglobin γ chain; *ALAS-E*: Aminolevulinate Synthase, Erythroid; *GPIIb*: Glycoprotein IIB; *RUNX1*: Runt-related transcription factor 1; *FLI1*: Friend leukemia integration 1 transcription factor; *MPL*: thrombopoietin receptor; *PAX5*: Paired Box 5; *EBF1*: Early B Cell Factor 1; *BLNK*: B Cell Linker. (**b**) Cells distribution across the different cell cycle phases analyzed by Propidium Iodide staining (PI). Dark gray: G2/M phase; light gray: S phase; white: G0/G1 phase. Arrows indicate significant reduction of the percentage of cells in S-phase.

Figure 3a (lower panel) shows that HEL cells, where growth arrest occurred more slowly compared to K562, indeed undergo a clear differentiation upon SOX6 overexpression, visible as a reduction in cell size. The analysis of prototypical lineage-specific genes confirmed that upon SOX6 overexpression, HEL and K562 cells increased the expression of erythroid marker genes. In cells with a more promiscuous erythro/megakaryocytic phenotype (MEG-01, SET-2, UKE1), the increase in erythroid genes expression occurred at the expenses of megakaryocytic-affiliated genes (i.e. *GPIIb*, *RUNX1*, *FLI1*, *MPL*). Surprisingly, in SUP-B15 cells, where the expression of erythroid-specific genes was -and remained- undetectable, SOX6 reduced the expression of the B-lineage master-regulator genes *EBF1*, *PAX5* and of the PAX5 downstream effector *BLNK*^28^ (Fig. 3a).

Consistently with previous observations, the most erythroid-biased cells, i.e. K562 and HEL (and to a lesser extent SET-2, which express erythroid genes at relatively high levels, not shown) responded to SOX6 by accumulating cells in G0/G1 at the expenses of the S phase (Fig. 3b), mimicking the cell cycle withdrawal steps accompanying late stages of erythroid differentiation^29^. This redistribution across cell cycle phases was not significant in MEG-01 and was absent in both UKE1 and SUP-B15 (Fig. 3b).

Taken together, these data indicate that within an erythroid context (HEL and K562) SOX6 induces terminal differentiation (Fig. 3) which justifies the block in cellular proliferation (Fig. 1e), in the absence of apoptosis (Fig. 2). On the other hand, in cells that are less biased towards the erythroid fate, SOX6 activates an apoptotic response leading to cell death (Fig. 2).

Collectively, the comparison of cell lines representing different hematological disorders reveals a dual effect elicited by SOX6, i.e. the induction of maturation (HEL and K562 cells) in erythroid cells and/or the induction of apoptosis in promiscuous (MEG-01, UKE1, SET-2) or non-erythroid cell types (B cells).

### SOX6 is downregulated in human hematological malignancies

These data collectively point to a broad anti-proliferative effect of SOX6, based on its ability to act via a dual mechanism, in a wide spectrum of cell types.

**Figure 4:**
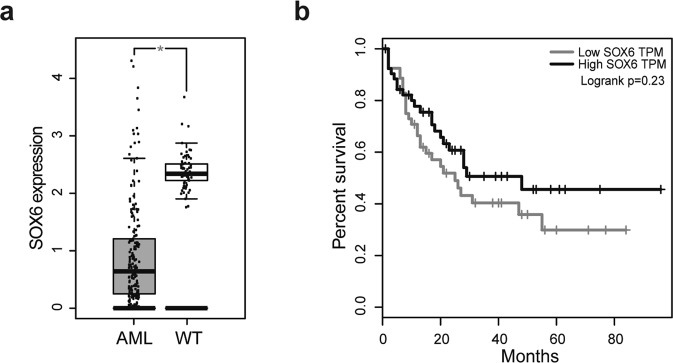
SOX6 is downregulated in AML patients. (**a**) GEPIA expression analysis of SOX6 in human AML patients. Gray box: 173 AML patients; white box: 70 healthy donors. (**b**) Kaplan–Meier curve comparing survival of AML patients with high -compared to low- SOX6 transcript level, based on GEPIA *p*-value = 0.23 was calculated with log-rank (Mantel–Cox) test.

This evidence prompted us to interrogate the GEPIA (Gene Expression Profiling Interactive Analysis) dataset^19^ to explore *SOX6* expression in normal vs. tumoral human hematological samples. Interestingly, *SOX6* expression was significantly reduced in a cohort of 173 AML patients (including few BCR-ABL1^+^ cases) (Fig. 4a). Moreover, the patients overall survival plot revealed a trend suggesting a direct correlation between low levels of *SOX6* and a poor prognosis (Fig. 4b).

Taken together, our data uncover an important anti-proliferative effect of SOX6 in a variety of hematological cancer cells, as a result of the induction of pro-differentiation, pro-apoptotic or both pathways. We believe that this is an important aspect in view of understanding the mechanism controlling the onset and/or the progression of cancer characterized by the loss of SOX6, and for the development of new treatment strategies.

## Methods

### Constructs

The *Sox6* murine cDNA (*SOX6*) was cloned in frame with a flag epitope immediately upstream to the IRES-EmeraldGFP (eGFP) or to the IRES-ΔNGFR cassette of the pHR SIN BX IR/EMW (originally derived from the pHR-SIN-CSGW lentiviral vector)^12^. The two packaging plasmids psPAX2 and pMD2.GVSVG were used to produce Lentiviral pseudo-particles in 293 T cells (www.lentiweb.com).

### Lentiviral production and transduction

Exponentially growing HEK-293T cells were transfected with the calcium-phosphate method with the above vectors plus the packaging plasmids psPAX2 and pMD2.G-VSVG to produce the lentiviral pseudo-particles. 72 h after transfection, the supernatant with recombinant virus particles was collected, filtered and centrifuged at 20,000 g for 8 hours at 4 °C. The viral pellet was re-suspended in 1XPBS and aliquoted at −80 °C. Lentiviruses were titrated on HEK-293T cells by measuring the percentage of eGFP- or of ΔNGFR-positive cells by Flow Cytometry (FC). The transduction of all the cell lines used in this study was performed overnight with a multiplicity of infection (MOI ≥ 30) by adding the viral particles re-suspended in 1X PBS directly to the cellular cultures.

### Whole protein extracts

Cells were harvested and centrifuged at 400 g for 5 minutes at room temperature. Pellets were washed twice in ice-cold 1X PBS (Euroclone) and re-suspended in RIPA buffer (20 mM TrisHCl pH 7.4; 150 mM NaCl; 5 mM EDTA; 0,3%TritonX) supplemented with proteases inhibitor cocktail (Roche). Lysis was performed for 30 minutes on ice. After centrifugation at 16000 g for 5 minutes at 4 °C, the supernatant containing protein extracts was retrieved.

### Immunoblotting analysis

Total extracts (15–30 μg/lane), resuspended in Laemmli buffer, were resolved by SDS/PAGE in a 10% acrylamide gel and blotted onto Hybond-ECL Nitrocellulose membrane (GE healthcare) under constant voltage at 100 V for 90 min at 4 °C (Biorad Transblot apparatus). Membranes were blocked for 1 hours at room temperature with Milk 5% in 1X TBS-T (Tris Buffered Saline, pH 7.6 and 0,1% Tween20) and incubated with the appropriate primary antibody diluted in Milk 5% 1X TBS-T overnight at 4°C. Membranes were washed three times in 1X TBS-T and incubated with the appropriate HRP-conjugated secondary antibody (in Milk 5% 1X TBS-T) for 1 hours at room temperature. After three washes in 1X TBS-T, antibodies binding was detected by ECL (Millipore). Antibodies used: anti-Flag F7425 (SIGMA) or ab125243 (abcam) and HRP-conjugated horse anti-mouse IgG #7076 (Cell Signaling), to detect the SOX6-FLAG protein; HRP-directly conjugated anti-beta actin #5125 (Cell Signaling) or anti-U2AF #U4758 (Sigma), to detect the loading control protein.

### *In vivo* induction of leukemia in mouse

B-ALL BCR-ABL1^+^ GFP^+^ cells were transduced with the SOX6-IRES-ΔNGFR vector or with the corresponding empty vector at a MOI = 50. 24 h later, cells were washed three times with 1X PBS and 10^6^ cells were injected into the tail vein of each C57BL/6 J recipient mouse. An aliquot of these cells was fixed in 4% paraformaldehyde, stained with APC Anti-human CD271 (#345108, BioLegend) for 15 minutes at 4 °C and analyzed for ΔNGFR positivity by flow cytometry. All the experiments and protocols were approved by the Ethical Committee on animal studies (OPBA: Organismo Preposto al Benessere Animale) of the University of Milano-Bicocca and were conducted according to the Italian legislation (approved protocol n°359/2016PR, authorized by the Italian Ministry of Health).

### Cell lines

For detailed description, please refer to https://web.expasy.org/cellosaurus/. Briefly: K562: erythroleukemia immortalized myelogenous cell line. MEG-01: human megakaryoblastic leukemic cell line. SUP-B15: Human B cell precursor leukemia. HEL: Human Erythroleukemic cell line. SET-2: human essential thrombocythemic. UKE1: human essential thrombocythemic cells. B-ALL BCR-ABL1^+^ GFP^+^: cell line established from Cdkn2a^−/−^ murine bone marrow cells infected with a pMIG retrovirus expressing a p190^BCR-ABL1^-GFP cassette (a gift from Prof. Ghysdael, Paris Diderot University). K562 and HEL cell lines were grown in RPMI medium (Lonza) supplemented with 10% of FBS (Fetal Bovine Serum) (Sigma); MEG-01 and SET-2 were grown in RPMI medium (Lonza) supplemented with 20% of FBS (Fetal Bovine Serum); UKE1 were grown in IMDM medium (EuroClone) supplemented with 10% FBS (Fetal Bovine Serum) (Sigma) and 10% Horse Serum (Invitrogen), 1uM di HydroCortisone (Sigma); SUP-B15 were grown in RPMI medium (Lonza) supplemented with 10% of FBS (Usa Origin Sigma); B-ALL BCR-ABL1^+^ GFP^+^ were grown in RPMI (Lonza) with 15% FBS (Sigma) and 7,5% NaHCO_3_. The medium of all the cell lines was supplemented with 100 μg/ml penicillin/streptomycin (EuroClone), 4mM L-glutamine (EuroClone). Cells were grown at an optimal concentration of 0,5 × 10^6^ cells/mL, in a humidified 5% CO_2_ atmosphere at 37 °C.

### RNA isolation and Real Time PCR

Total RNA from ≥10^6^ of all different cell lines and experimental condition were purified with TRIzol Reagent (Applied Biosystem), treated with RQ1 DNase (Promega) for 30 minutes at 37 °C and retrotranscribed (High Capacity cDNA Reverse Transcription Kit, Applied Biosystem). Negative control reactions (without Reverse Transcriptase) gave no PCR amplification. Real time analysis was performed using ABI Prism 7500, (Applied Biosystems). Primers were designed to amplify 150 to 300 bp amplicons, spanning an exon-exon junction when possible, on the basis of sequences from the Ensembl database (http://www.ensembl.org). Samples from each experiment were analyzed in triplicate. Specific PCR product accumulation was monitored by using SsoAdvanced™ Universal SYBR® Green Supermix (Bio-Rad) fluorescent dye in 12-μl reaction volume. Dissociation curves confirmed the homogeneity of PCR products. All primers are available upon request.

### Apoptosis Assay

Apoptosis assays were carried out by using AnnexinV (AnnV) and 7-Amino-Actinomycin D (7-AAD) (BD Biosciences). 2,5 × 10^5^ cells were washed twice with cold 1X PBS (Euroclone) and then resuspend in 1X Annexin Binding Buffer (10 mM Hepes pH 7.4), 140 mM NaCl, 2,5 mM CaCl_2_) with 5 μl of PE Annexin V (BD Biosciences) or APC Annexin V (Biolegend) and 5 μl 7-AAD in a final volume of 100 μL. Cells were gently vortexed and incubated for 15 minutes at RT (25 °C) in the dark. Finally, samples were diluited with 400 μl of 1X Annexin Binding Buffer and analyzed by flow cytometry within 1 hour (Becton-Dickinson FACS Calibur or Beckman-Coulter CytoFLEX S). Data were analyzed with the Summit Software 4.3 or CytExpert Software.

### Propidium Iodide (PI) Staininig for cell cycle analysis

Cells, washed twice with cold 1X PBS (Euroclone), were resuspend in 1% paraformaldehyde for 45 minutes. After that, the same volume 1X PBS with Triton 0,2% (Sigma) was added for 15′ minutes. Cells were then centrifuged and resupended in 250 μl of PBS with 0.1 mg/ml PI (Sigma) and 100 μg/mL RNAse (Sigma), vortexed for 5 seconds and incubated at room temperature for at least 1 hour in the dark. Samples were analyzed by flow cytometry within 1 hour (Becton-Dickinson FACS Calibur or Beckman-Coulter CytoFLEX S). Data were analyzed with the Summit Software 4.3 or CytExpert Software.

### Cells staining

1,5 × 10^5^ cells were resuspended in 1X PBS with 0,1% BSA and cytospun at 300 rpm for 2 minutes at room temperature. Cells were then fixed with 10% cold Methanol and stained by using the Ematoxylin-Eosin staining kit Diff-Quick dyes (Medium Diagnostics) according to the manufacturer’s protocol.

### Statistics

All continuous variables have been tested for normality by using the Shapiro-Wilk test. Experiments on cell lines were analyzed by using a test for paired data while for the *in vivo* experiments data were analyzed through tests for unpaired data. All tests performed were two-tailed. Statistical analysis was performed by using GraphPad Prism, version 6.0. Data are expressed as mean ± SEM of n ≥ 3 replicates.

### Ethical Statement

All the experiments in mice were conducted according to the Italian legislation (protocol n°359/2016PR, authorized by the Italian Ministry of Health).

## Supplementary information

Supplementary information

## Data Availability

No datasets were generated in the current study. Figure 4 is based on GEPIA data (Gene Expression Profiling Interactive Analysis, -http://gepia.cancer-pku.cn/-, ref.^19^).
