# Supplementary material for: SOX6 blocks the proliferation of BCR-ABL1+ and JAK2V617F+ leukemic cells
Source: Sci Rep. 2019 Mar 4;9:3388. doi: 10.1038/s41598-019-39926-4 (PMC6399316; doi:10.1038/s41598-019-39926-4)
Supplement: Supplementary file 1 — Supplementary information [file 41598_2019_39926_MOESM1_ESM.pdf]

SOX6 blocks the proliferation of BCR-ABL1^{+} and JAK2V617F^{+} leukemic cells

Gloria Barbarani, Cristina Fugazza, Silvia Maria Luisa Barabino and Antonella Ronchi

SUPPLEMENTAL FIGURES:

Supplemental figure 1

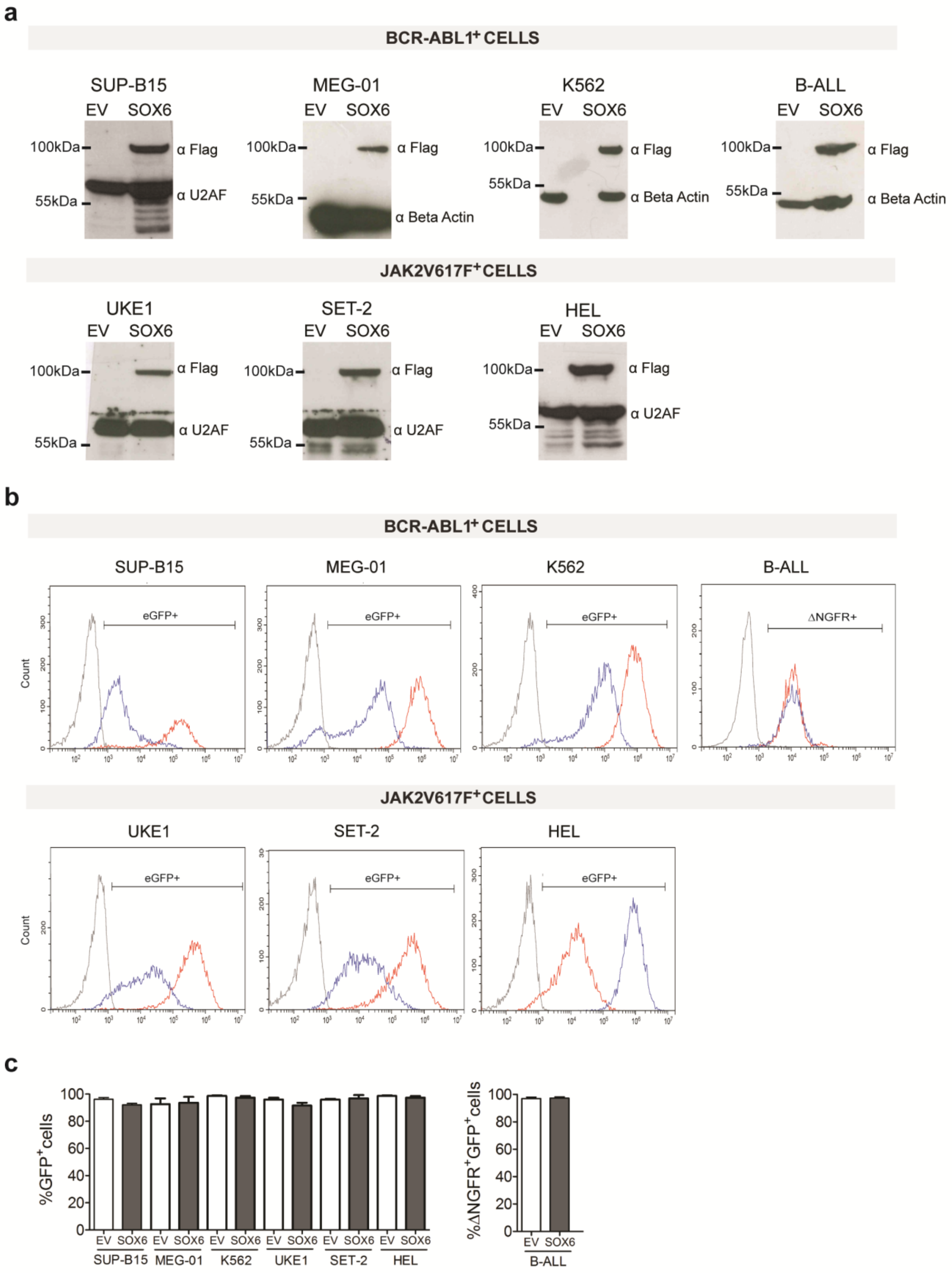

Supplemental Figure 1: a) Representative Western Blots showing the SOX6 exogenous overexpression in the different cell lines. An anti-Flag antibody is used to detect the SOX6-Flag protein. Beta-Actin or U2AF are used as a loading control. b) Representative plots from flow cytometry analysis performed on the same cells. To assess the efficiency of transduction, eGFP or APC-CD271 (to detect ∆NGFR protein in B-ALL) have been used. Grey: un-transduced cells. Red: EV-transduced cells. Blue: SOX6-transduced cells. c) All cell lines were >97% transduced, as shown by the histograms representing the average of eGFP or ΔNGFR positivity in each cell type.

Supplemental figure 2

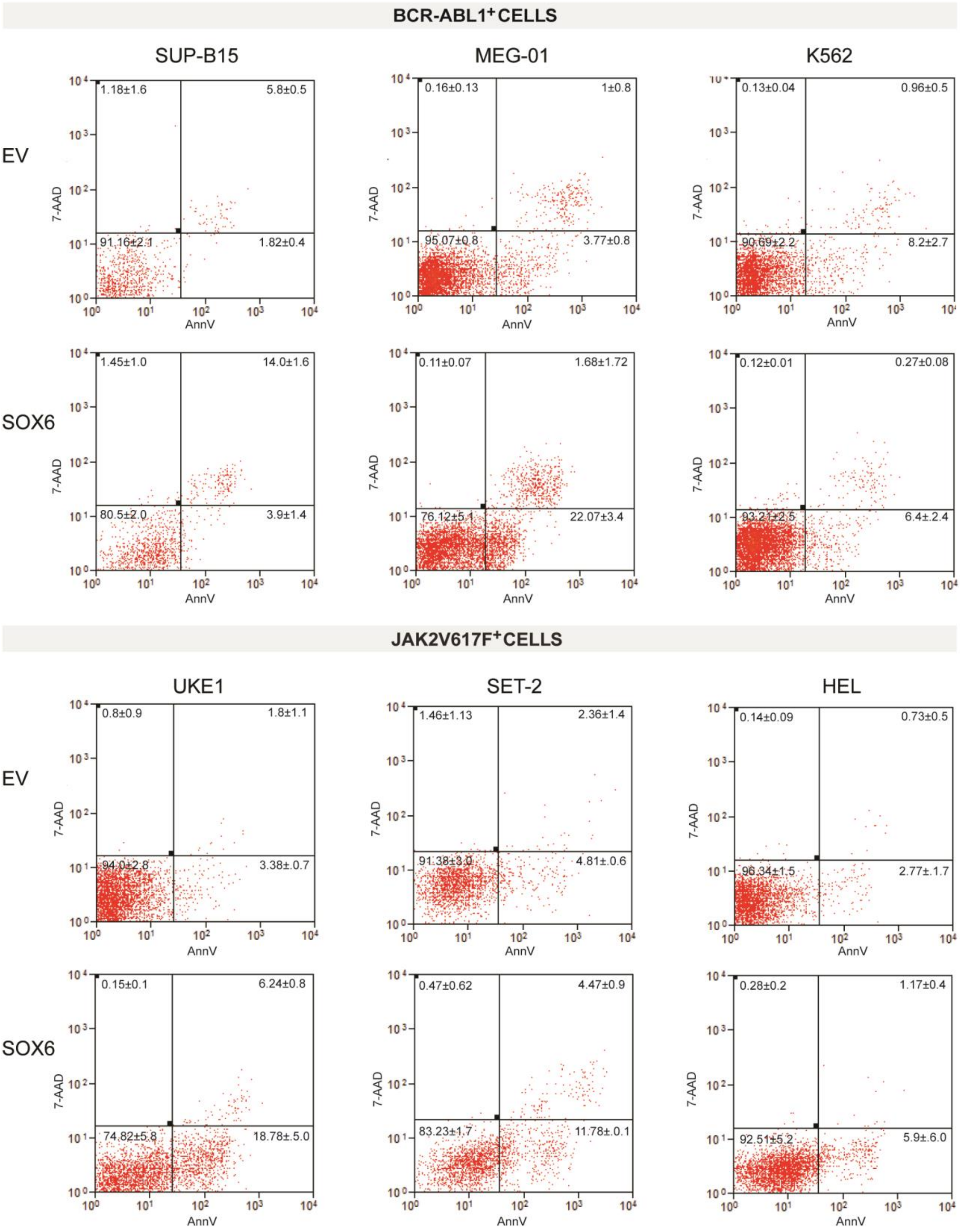

Supplemental Figure 2:

Representative plots of the apoptosis analysis performed by flow cytometry for each cell type. Apoptosis was assessed on gated GFP^+ cells by APC-AnnexinV and 7-AAD staining. Each quadrant contains the average±SD values relative n≥3 independent experiments. EV: cells transduced with the empty vector. SOX6: cells transduced with the SOX6-expressing vector.
